# Influence of physically demanding occupations on the development of osteoarthritis of the hip: a systematic review

**DOI:** 10.1186/s12995-022-00358-y

**Published:** 2022-08-24

**Authors:** Susanne Unverzagt, Ulrich Bolm-Audorff, Thomas Frese, Julia Hechtl, Falk Liebers, Konstantin Moser, Andreas Seidler, Johannes Weyer, Annekatrin Bergmann

**Affiliations:** 1 Institute of General Practice and Family Medicine, Martin Luther University Halle-Wittenberg, Magdeburger Str. 8, 06112 Halle (Saale), Germany; 2 Department for Occupational Safety and the Environment, Regional Authority Darmstadt, Wilhelminenstraße 1 - 3, 64283 Darmstadt, Germany; 3 Department of Occupational Medicine, Martin Luther University Halle-Wittenberg, Magdeburger Str. 20, 06112 Halle (Saale), Germany; 4 Division 3 Work and Health, Unit 3.1 Prevention of Work-related Diseases, Federal Institute for Occupational Safety and Health (BAuA), Friedrich-Henkel-Weg 1-25, 44149 Dortmund, Germany; 5 Institute and Polyclinic for Occupational and Social Medicine, Technical University of Dresden, Faculty of Medicine, Löscherstraße 18, 01309 Dresden, Germany

**Keywords:** Occupational, Hip, Osteoarthritis, Workplaces, Musculoskeletal

## Abstract

**Background:**

Hip osteoarthritis (HOA) is a disabling disease affecting around 33 million people worldwide. People of working age and the elderly are at increased risk of developing HOA and the disease is associated with high costs at individual and societal levels due to sick leaves, job loss, total hip replacements and disability pension. This systematic review evaluated the influence of physically demanding occupations on the development of HOA in men.

**Methods:**

Cohort studies, case–control studies and cross-sectional studies with publications in English or German, which assessed the association between exposure to physically demanding occupations and development of HOA, were searched in electronic databases (Medline, Embase, HSE-Line, Cochrane Library) and conference abstracts from 1990 until May 2020. We assessed the methodological quality of selected studies, interpreted all relative effect estimators as relative risks (RRs) and meta-analytically reviewed the effects of occupations with high physical workloads. All steps are based on a study protocol published in PROSPERO (CRD42015016894).

**Results:**

Seven cohort studies and six case–control studies were included. An elevated risk to develop HOA was shown for six physically demanding occupational groups. Working in agriculture including fishery and forestry and food production doubles the risk of HOA. Construction, metal working and sales as well as exposure to whole body vibration while driving vehicles increases the risk by roughly 50 to 60%. Unskilled or basic level workers, who were frequently exposed to repetitive heavy manual work, had nearly a doubled risk (RR 1.89 95%CI: 1.29 to 2.77) compared to workers with lower exposure.

**Conclusions:**

Existing studies state an association between various occupations with high physical workload and an increased risk of developing HOA. High Physical workloads include including lifting and carrying heavy loads, demanding postures, repetitive activities, long standing and running, as well as exposure to body vibrations. Occupational prevention and early detection as well as individual health promotion strategies should place their focus on reducing the impact of high physical strain at work sites.

**Supplementary Information:**

The online version contains supplementary material available at 10.1186/s12995-022-00358-y.

## Background

Hip osteoarthritis (HOA) is a disabling disease characterized by pathologic changes of the whole hip joint including the articular cartilage, subchondral bone, and the synovial joint lining [1, 2]. In European studies, HOA prevalence estimates range from 2 to 9% for people under 75 years of age depending on the region and diagnostic criteria [3–5]. The Global Burden of Disease Study 2019 reported lower prevalence estimates for people over the age of 25 with 1.95% (95%-CI 1.48 to 2.49) in Western Europe and 1.01% (95%-CI 0.77 to 1.27) in Eastern Europe [3]. HOA incidence increases at the age of 40–50, which indicates an increased risk of developing HOA for people of working age and the elderly. This is associated with high costs due to necessary medical treatments, sick leaves, job loss or disability pensions [6]. This substantial health burden might be further increased by factors such as willingness of patients to seek care, awareness of primary care physicians, aging, increasing obesity and more frequently occurring joint injuries [1, 2, 7]. On the other hand, changes in occupational exposures, the development of special preventive strategies and technical equipment may reduce this burden [8 9]. Other confirmed risk factors for developing HOA are genetic pathologies and congenital deformit [1–13].

The American College of Rheumatology has established diagnostic criteria for HOA, which are the most frequently used internationally [14]. These include hip pain, restricted internal hip rotation and radiographic changes such as joint space narrowing and marginal femoral or acetabular osteophytes. The Kellgren and Lawrence score and other joint functioning scores are also considered for diagnosis of HOA [15, 16].

Various reviews demonstrate a positive association between long-term lifting and carrying of heavy loads, or physically demanding work in general, and the risk of developing of HOA [17–20]. Thus, in Germany, HOA has been defined in 2020 as an occupational disease for workers lifting heavy loads [21] with a cumulative load of at least 9.500 tons in their working life or ≥ 20 kg handled > 10 times per day. However, HOA is not listed in the European schedule of occupational diseases [22, 23].

We wanted to check whether only occupations associated with lifting and carrying heavy loads have an increased risk of developing HOA, or whether other physically demanding occupations with demanding postures, repetitive activities, long standing and running or body vibrations may also increase the risk of HOA. The aim of this systematic review was to identify studies and appraise and synthesize their results on developing HOA due to specific occupations. The hypothesis was that an increased risk of developing HOA would be observed in occupational sectors with high physical workloads compared to other occupational sectors. This systematic literature review was based on a sensitive search string and concentrated on the exposure of men in physically demanding occupations. Due to the high number of occupations, substantial differences in work exposures for men and women and job sector segregation between the sexes [24], we decided to summarize and discuss the results for women, including gender-specific differences and consequences, in a separate manuscript.

## Methods

This systematic review was registered a priori in PROSPERO (CRD42015016894) and follows the PRISMA regulations [25].

### Systematic search

We systematically searched cohort studies, case–control studies and cross-sectional studies that assessed the association between exposure to occupational workload or occupations with known high physical workload and development of HOA in February 2015 and published the results on the association between lifting and carrying heavy loads or other demanding work on the risk of HOA [17] and added searches in 2018 and 2020 on the topic of this review. We searched Medline (Ovid) (Supplementary Table S1), Embase (Supplementary Table S2), Cochrane Library (Supplementary Table S3), CINAHL (Supplementary Table S4) and the Health and Safety Executive (HSE)-Line (Supplementary Table S5) with a last update of the search conducted in May 2020. We manually checked the tables of contents of occupational medicine journals (Supplementary Table S6) and checked the abstracts of annual meetings of international orthopaedic conferences (International Epidemiology in Occupational Health (EPICOH), American Occupational Health Conference (AOHC) and the American Orthopaedic Association (AOA)) for additional studies until 2020. Embase was searched in February 2015. The search strategy was based on a combination of controlled vocabulary and key words to describe the association between occupational exposure and the development of HOA. All references were imported to a bibliographic reference management program (Endnote).

### Inclusion and exclusion criteria

Criteria summarized in Table 1 were applied to each identified reference to determine eligibility for this systematic review.

**Table 1:** Inclusion criteria for studies

Population	Adult men (≥ 18 years at diagnosis)
Exposure	Former or current employment in occupational groups with known heavy physically demanding strain where results on the association between occupational strain and development of HOA were reported in at least three studies
Comparison	Occupational groups without heavy occupational workload
Endpoints	Diagnosis of HOA due to occupational strain based on •clinical and radiological criteria (American College of Rheumatology criteria [14] or radiological scoring systems according to Kellgren and Lawrence [16]) •total hip replacement subscales measuring hip pain, stiffness or reduced physical function •hip pain •reported cases with the diagnosis HOA in register data without further information including resulting disability, pension or sick leave Exclusion of studies with HOA due to non-occupational strains (e.g., due to sport, hip dysplasia or other hip deformities)
Study design	Full-text publications in German or English language from 1990 until today of •cohort studies •case–control studies or analysis of data from registry data •cross-sectional studies with relevant data of an exposure to occupations for ≥ 10 years to assure a causal relationship

### Study selection

Two independent reviewers checked titles and abstracts of all references identified from different sources of our systematic search, read full text publications of potentially eligible studies, extracted data and assessed the quality of included studies. In case of disagreement, a third reviewer was consulted to achieve consensus.

### Data extraction and quality assessment

We extracted information to characterize the researched studies (design, country and time of recruitment), study population (inclusion criteria, number of participants, age and sex), exposure and reference groups (including levels of exposure with their duration and intensity) and outcome (with precise diagnostic criteria). To describe the investigated association, we extracted numbers of participants and events per group and adjusted effect sizes with their 95% confidence intervals (95%-CI) for men and women.

Quality assessment was based on criteria recently used in Bergmann et al. 2017 [17]. These criteria applied the Newcastle–Ottawa Quality Assessment Scale [26] and the Cochrane Handbook [27] to our research question. The assessment criteria were separately developed for case–control and cohort studies. For cohort studies, we judged the representative selection of exposed and non-exposed participants, validity and accuracy of exposure acquisition and assessment of outcomes and the methods to ensure comparability of exposed and non-exposed groups. For case–control studies, we judged the selection, validity and accuracy of exposure and methods to ensure comparability of cases and controls. Best judgement resulted in 19 points for cohort studies and 15 points for case–control studies.

### Data synthesis

The association of occupational risks and development of HOA was assessed with all effect measures to compare the risk of occupations with heavy physical strain to non-exposed reference groups. Due to the low prevalence of HOA, different reported effect measures (odds ratios, relative risks, hazard ratios, standardised hospitalisation ratios) do not differ widely and were interpreted as relative risks (RR). If more than one effect estimator was reported in one study, we pooled comparable results from studies, which corresponded best with our research question. This estimator was based on diagnostic criteria with the best validity according to Bergmann et al. 2017 [17] and the highest or longest exposure with a sufficient sample size.

The effect estimators of different studies and their 95%-CI were synthesized with the random effects model using RevMan 5.3 [28] due to differences in measurement of exposure, outcome, study design and effect measures. Reported RRs greater than 1 are considered as a higher risk in occupations with heavy physical strain compared to the reference group. We judged the consistency of results of different studies based on the I^2^ value and interpreted heterogeneity as small if I^2^ < 30%, moderate (30 to 60%) or substantial (I^2^ > 60%). We did not discuss the pooled results in cases of substantial heterogeneity between study results, different conclusions of the studies or clinical heterogeneity in severity of physical demands. To investigate clinical heterogeneity between treatment effects of individual studies, we calculated subgroup analyses for differences in exposure, criteria to diagnose HOA and study design.

## Results

**Fig. 1:**
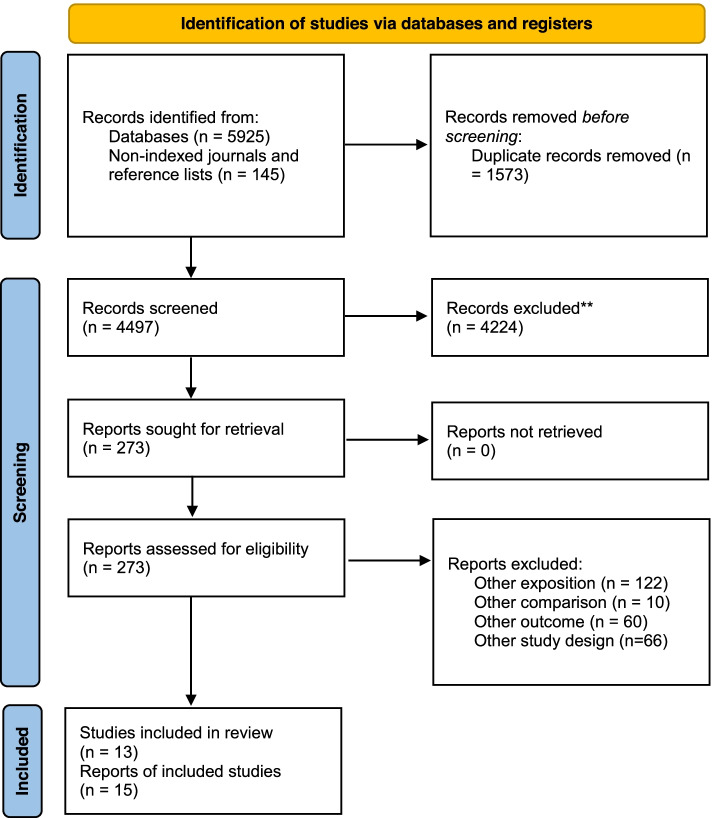
Flow chart [44] to describe identification and selection of included studies

We identified a total of 6070 records and read 273 potentially relevant reports. After exclusion of all reports that did not fulfil our inclusion criteria, we included a total of 13 eligible studies including 7 cohort studies [29–37] and 6 case–control studies [38–43] with a total of 15 publications (Fig. 1). Cross-sectional studies with an exposure to occupations of at least 10 years to ensure the association between occupational exposure and the development of HOA were not identified.

### Participants

All eligible studies were conducted in Europe, predominantly in Scandinavian countries (Sweden, Denmark, Finland, and Iceland), two studies in the UK and one in Germany. Most studies included working age participants with a mean or median age of over 36 years at time of diagnosis. The proportions of men showed a broad variation across occupational groups. Most studies recruited more men and four studies [38, 39, 42, 43] recruited only men (Supplementary Tables S7 and S8).

### Exposure

Information on occupation was based on registry data [29, 31–37] in Scandinavian countries, occupational titles [32] and interviews [38, 39, 43] or questionnaires [40–42] on occupational history. Some studies summarized different occupational groups, others were more specific and differentiated between degrees or periods of exposure. This review focuses on results on the risk to develop HOA in physically demanding occupational groups traditionally dominated by men (farming, construction, driving, metal work, storage and transportation).

We identified nine occupational sectors with physically demanding workloads and resulting high risk to develop HOA. These sectors considered occupations in the following:

•agriculture, fishery or forestry (12 studies): Three of these studies [38, 42, 43] differentiated between exposure lengths. Studies reported results on the exposure of farmers or summarized farmers and forest workers [40, 43] farmers and agricultural workers [38] or agricultural and fishery workers [34] (Supplementary Table S9).
•construction (9 studies): Five studies reported the risk for construction workers [29, 31, 37, 40, 43], three studies summarized exposure of comparably demanding occupations [34, 39, 41] and one study reported more specialized occupations [33] (Supplementary Table S10).
•driving vehicles with exposure to whole body vibration (8 studies): Two studies reported on the risk of any occupation with exposure to whole body vibration [33, 40], others reported results for professional motorists [31, 34, 39] or differentiated between driving different vehicles [36, 37, 42] (Supplementary Table S11).
•metal work (6 studies): Of these, three studies reported the risk of general occupations in metal work [31, 40, 43], others summarized metal and machinery workers [34] or reported more specific information on sheet-metal workers [33] and furnace men, smiths and workers doing metal processing [37] (Supplementary Table S12).
•sales (5 studies): Of these, four studies reported the risk of working as service or shop worker [34–41], whereas one [37] summarized very heterogeneous demanding occupations for store and warehouse workers (Supplementary Table S13).
•gastronomy (4 studies): All of these studies summarized different occupations with comparable exposure in gastronomy and hotels [34, 36, 37, 40] (Supplementary Table S14).
•food production (4 studies): Three studies summarized comparable demanding occupations [37, 40, 43], whereas one study [36] reported the risk of less demanding occupations as baker, pastry cook or confectionery maker (Supplementary Table S15).
•storage transportation (3 studies): All studies investigated the exposure of postmen [38, 41, 44], one study added results for storage and transport workers (Supplementary Table S16).
•health care (3 studies): Studies reported results for health-care assistants [29], medical doctors [36] and one study reported combined results for nurses and environmental officers [34] (Supplementary Table S17).

Finally, three studies [34, 37, 41] investigated the risk of HOA of men in unskilled or basic level labours in comparison to managers, professionals or low-exposure blue-collar workers (Supplementary Table S18).

### Diagnosis

Follow-up periods between exposure and diagnoses are mainly available from cohort studies and ranged from 3 to 28 years.

Various criteria were used for the diagnosis of HOA:

•Disability pension or sick leave due to HOA [31, 34, 43]
•Implantation of a total hip replacement (THR) [33, 36, 41]
•Radiological diagnostic criteria or THR [39, 42]
•Clinical or radiological diagnostic criteria [29, 35, 37, 38, 40]
•Disability pension due to low back disorders [43]

A high validity of the diagnosis of HOA was assumed for disability pension due to HOA, THR and radiological imaging compared to a clinical diagnosis and back disorders.

### Quality assessment

#### Cohort studies

The seven cohort studies scored 12 to 15 points on a scale ranging from 0 to 19 points. No study could provide accurate and reliable quantitative data on exposure such as frequency or duration of occupation. All studies adjusted results for age; three used other important confounders (body mass index or education and physical workload factors) [31, 33, 34] (Supplementary Table S7).

#### Case–control studies

The six case–control studies scored between 5 to 10 points on a scale of 0 to 15. Three studies received less than 3 out of 6 points for the selection of the study population [38–40] due to deficits in the representativeness of the cases and the selection of controls. Two studies [40, 41] had a low response rate. Only one study received full points for an adequate case definition by consideration of people with a THR [41] and one study received no points due to the low validity of diagnostic criteria [43]. Only one studies excluded pre-existing HOA al the baseline [35]. Only two studies received points for including a long enough follow-up of more than 10 years for the endpoint to occur [42, 43]. Four studies were downgraded due to low validity of data acquisition [39–42] and two studies [40, 43] were downgraded because some comparative hypotheses mentioned in the methods section were not reported with quantifiable data. Two studies [38, 41] adjusted their results for age and body weight (Supplementary Table S8).

### Study results

#### Agriculture, fishery or forestry (12 studies)

**Fig. 2:**
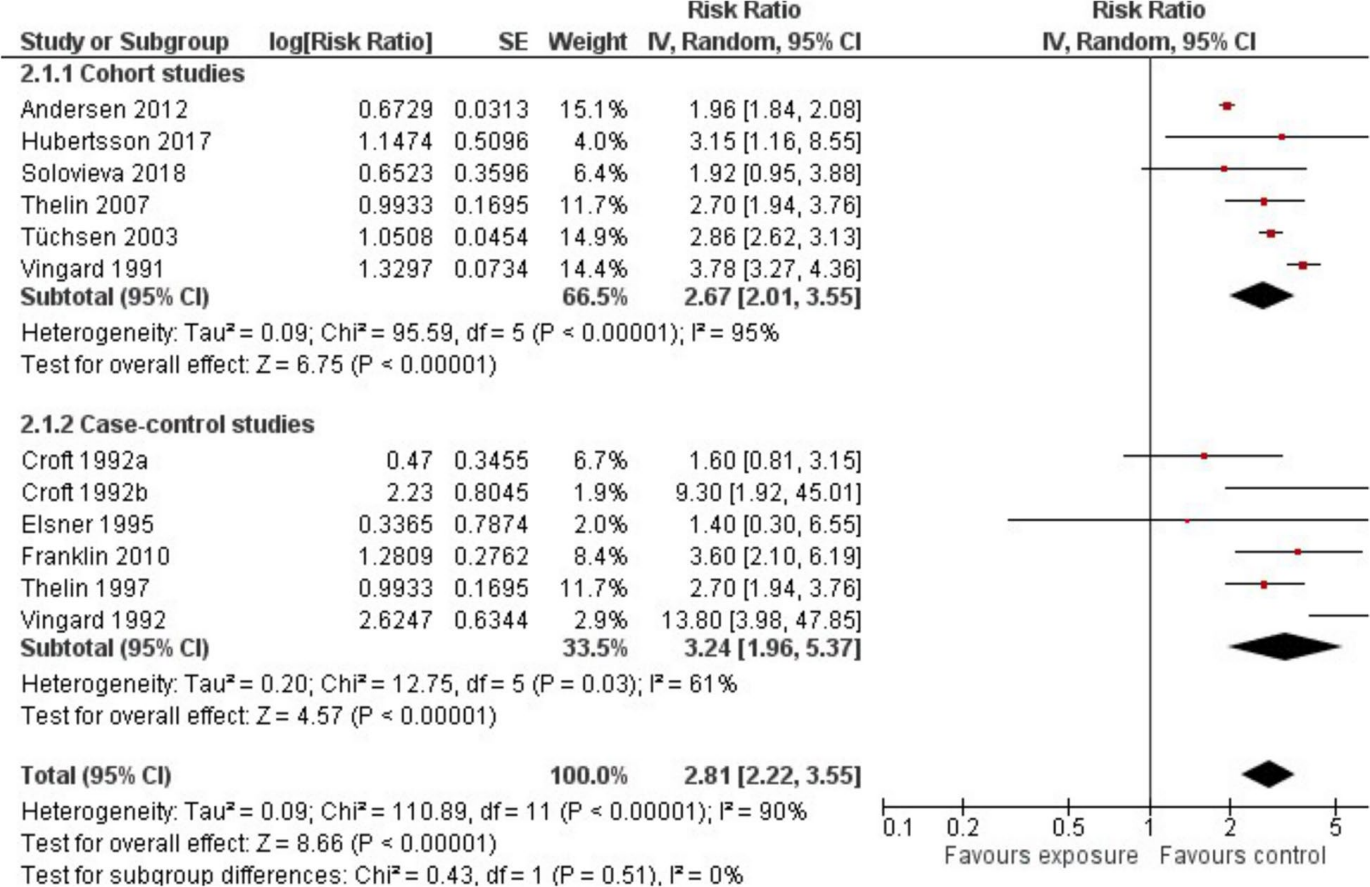
Risk of developing HOA owing to occupations in agriculture, fishery or forestry: Comparison of exposed and non-exposed control groups

Out of around 273,300 men with occupations in agriculture or forestry, 4,732 were diagnosed with HOA. All studies stated an elevated risk of developing HOA with a pooled RR of 2.81 (95%-CI 2.22 to 3.55) with substantial heterogeneity (I^2^ = 90%) between results of different studies (Fig. 2). A possible explanation for this heterogeneity might be the increasing relative risk of HOA with increasing numbers of years farming as shown in two studies [38, 42, 45] (Supplemental Table S9).

#### Construction worker (9 studies)

**Fig. 3:**
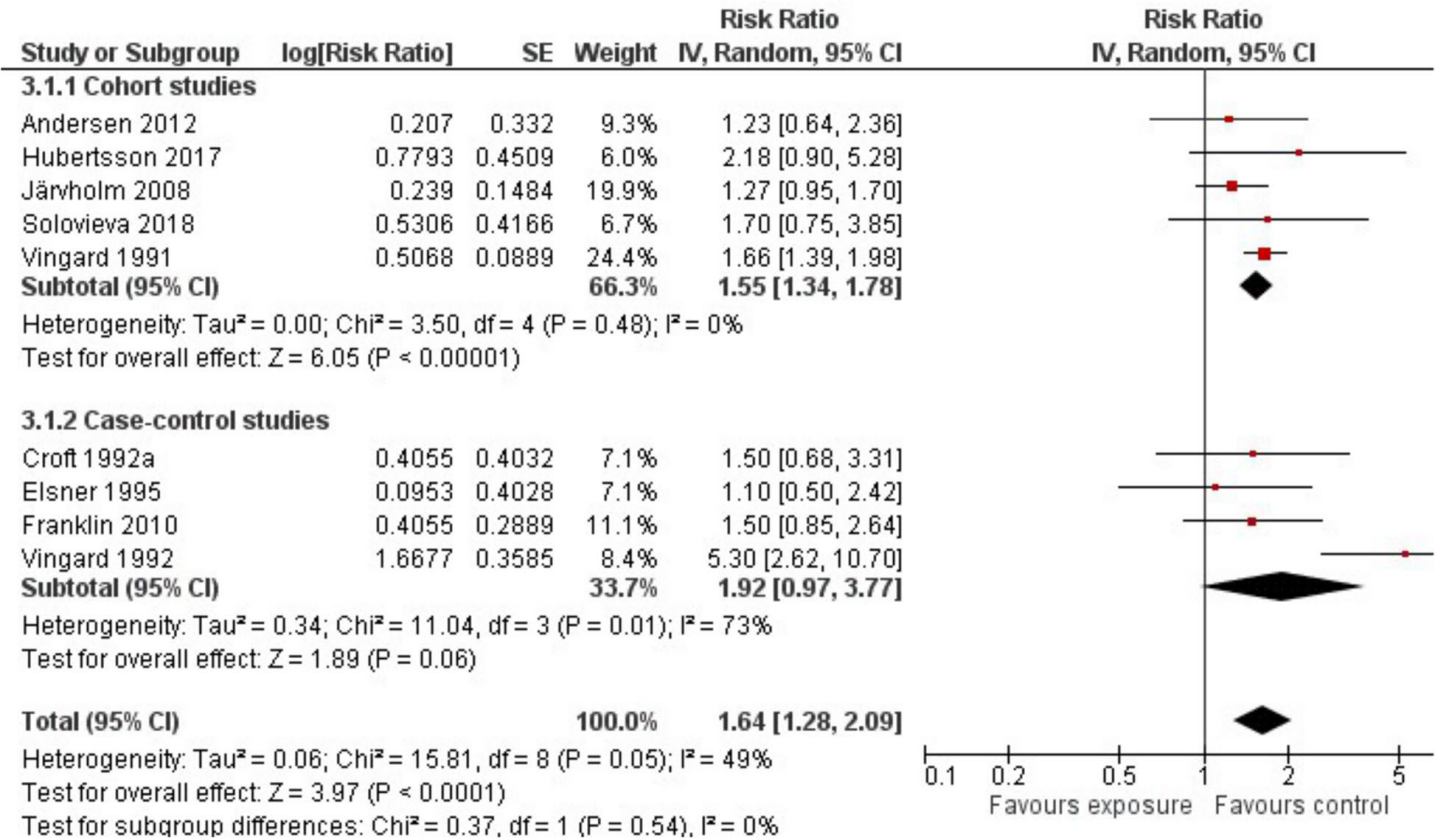
Risk of developing HOA as a construction worker: Comparison of exposed and non-exposed control groups

Out of more than 581,000 construction workers, 3,736 were diagnosed with HOA. The results of all studies stated an elevated risk of developing HOA. Summarising the results of all studies, we stated that the risk of construction workers to develop HOA is increased by 64% (RR 1.64; 95%-CI 1.28 to 2.09) compared to non-exposed men with a moderate heterogeneity (I^2^ = 49%) between studies (Fig. 3).

#### Driving vehicles with whole body vibration (8 studies)

**Fig. 4:**
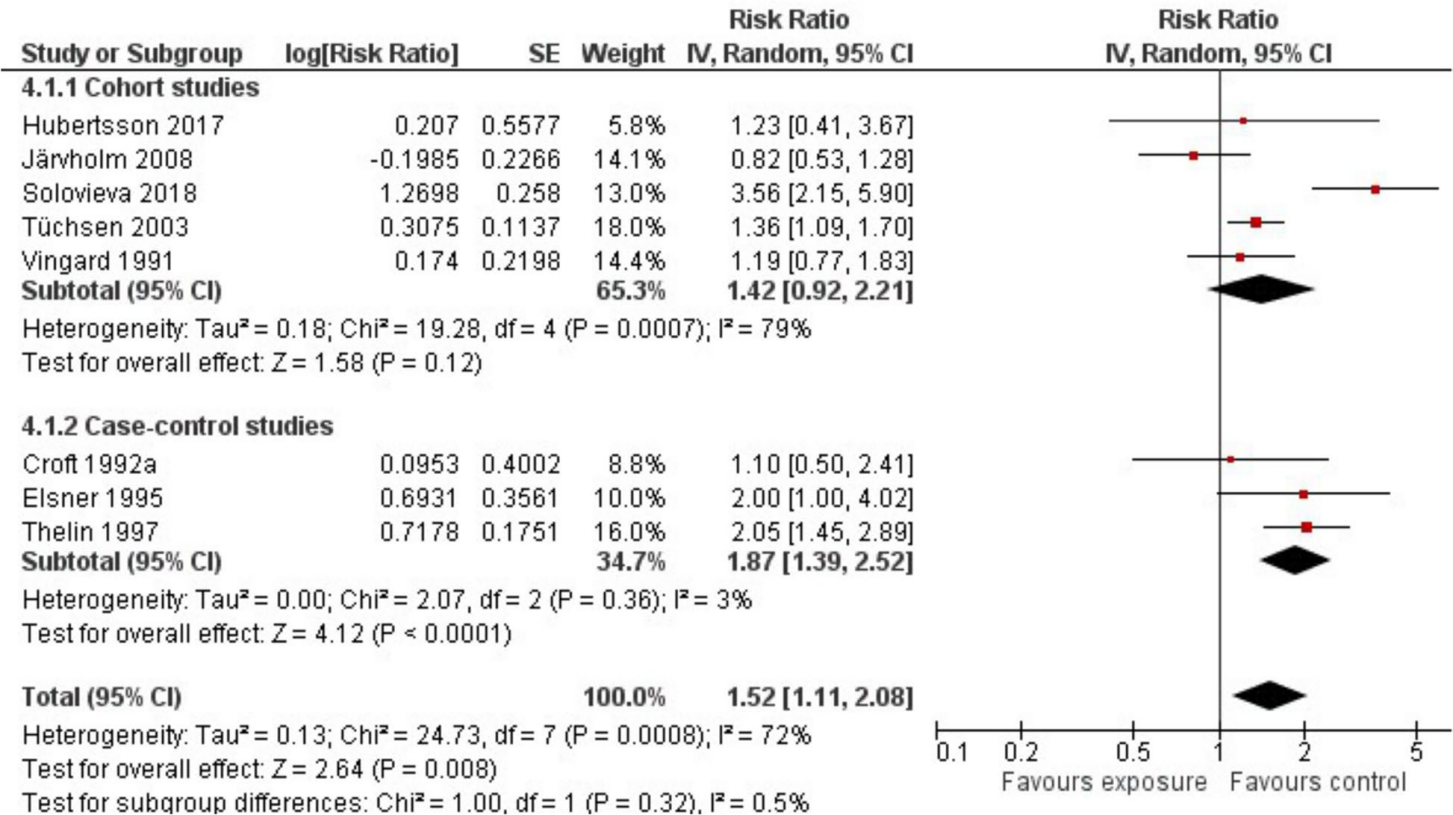
Risk of developing HOA in occupations with whole body vibrations due to driving vehicles: Comparison of exposed and non-exposed control groups

Out of more than 93,418 drivers, 410 (most of them driving different vehicles with whole body vibration) were diagnosed with HOA. The results of 7 out of 8 studies stated an elevated risk with a pooled RR of 1.52 (95%-CI 1.11 to 2.08) and a substantial heterogeneity (I^2^ = 72%) between results of different studies (Fig. 4). The heterogeneity might be explained by the exposure to very different vehicles and resulting vibrations (Supplementary Table S11).

#### Metal work (6 studies)

Out of more than 77,000 metal workers, 299 were diagnosed with HOA. The results of all studies stated an elevated risk to develop HOA. Summarising the results of all studies, we stated an increased risk of 54% (RR 1.54; 95%-CI 1.21 to 1.97) for metal workers compared to non-exposed men with moderate heterogeneity between results (I^2^ = 37%) (Table 2).

**Table 2:** Effect sizes of occupational exposure to develop HOA among different subgroups

Occupation	Subgroup	Number of studies	RR (95%-CI)	Hetero- geneity (I^2^)	Subgroup differences
**Agriculture and forestry**	**All studies**	**12**	**2.81 (2.22–3.55)**^a^	**90%**	
	Disability pension due to HOA	3	3.96 (1.36–11.51)^a^	73%	*p *= 0.72
	THR, no information on disability pension reported	3	2.41 (1.45–4.00)^a^	91%	
	Diagnosis, but no THR	6	2.60 (1.81–3.72)^a^	92%	
	Cohort studies	6	2.67 (2.01–3.55)^a^	95%	*p* = 0.51
	Case–control studies	6	3.24 (1.96–5.37)^a^	61%	
**Construction worker**	**All studies**	**9**	**1.64 (1.28–2.09)**	**49%**	
	Disability pension due to HOA	3	2.79 (1.36–5.72)	59%	*p* = 0.12
	THR, no information on disability pension reported	2	1.31 (1.02–1.70)	38%	
	Diagnosis, but no THR	4	1.60 (1.36–1.87)	0%	
	Cohort studies	5	1.55 (1.34–1.78)	0%	*p* = 0.54
	Case–control studies	4	1.92 (0.97–3.77)^a^	73%	
**Driving vehicles with whole body vibration**	**All studies**	**8**	**1.52 (1.11–2.08)**^a^	**72%**	
	Disability pension due to HOA	2	2.35 (0.85–6.49)^a^	67%	*p* = 0.18
	THR, no information on disability pension reported	3	1.12 (0.78–1.59)	51%	
	Diagnosis, but no THR	3	1.68 (1.15–2.45)	50%	
	Cohort studies	5	1.42 (0.92–2.21)^a^	79%	*p* = 0.32
	Case–control studies	3	1.87 (1.39–2.52)	3%	
**Metal work**	**All studies**	**6**	**1.54 (1.21–1.97)**	**37%**	
	Disability pension due to HOA	3	1.83 (1.15–2.90)	0%	*p* = 0.28
	THR, no information on disability pension reported	1	1.10 (0.67–1.80)	-	
	Diagnosis, but no THR	2	1.72 (1.10–2.68)	22%	
	Cohort studies	4	1.42 (1.08–1.87)	0%	*p* = 0.21
	Case–Control studies	2	2.09 (1.23–3.56)	0%	
**Sales**	**All studies**	**5**	**1.50 (1.01–2.24)**^b^	**38%**	
	Comparable physical demanding occupations	4	1.50 (0.82–2.75)	51%	*p* = 0.83
	Heterogeneous physical demanding occupations	1	1.39 (0.95–2.03)	-	
	Disability pension due to HOA	1	2.35 (1.19–4.66)	-	*p* = 0.33
	THR, no information on disability pension reported	2	1.77 (1.00–3.16)	0%	
	Diagnosis, but no THR	2	0.96 (0.37–2.51)^c^	65%	
	Cohort studies	2	1.67 (1.02–2.72)	42%	*p* = 0.51
	Case–Control Studies	3	1.21 (0.54–2.70)	56%	
**Gastronomy**	**All studies**	**4**	**1.44 (0.78–2.66)**^c^	**63%**	
	Cohort studies	3	1.70 (0.92–3.15)^a^	63%	*p* = 0.13
	Case–Control Studies	1	0.60 (0.18–2.02)	-	
**Storage and transportation**	**All studies**	**3**	**2.09 (0.88–4.96)**	**0%**	
	Cohort study	1	0.92 (0.12–6.99)	-	*p* = 0.51
	Case–Control-Studies	2	2.50 (0.96–6.52)	0%	
**Food production**	**All studies**	**4**	**2.13 (1.53–2.95)**^b^	**0%**	
	Comparable physical demanding occupations	3	2.22 (1.39–3.54)	0%	*P* = 1.00
	Less physical demanding occupations in construction	1	2.04 (1.28–3.24)	-	
	Cohort study	2	2.10 (1.48–2.96)^b^	0%	*p* = 0.84
	Case–Control-Studies	2	2.40 (0.64–8.97)	37%	
**Health care**	**All studies** (only cohort studies)	**3**	**1.21 (0.51–2.88)**^c^	**88%**	
**Unskilled or basic level labours**	**All studies**	**3**	**1.89 (1.29–2.77)**	**34%**	
	Cohort studies	2	2.15 (1.31–3.54)	46%	*p* = 0.28
	Case–Control Studies	1	1.4 (0.77–2.56)	-	

*CI* confidence interval, *HOA* hip osteoarthritis, *RR* relative risk, *THR* total hip replacement

Pooling not useful due to substantial heterogeneity between results of individual studies (^a^), clinical heterogeneity in severity of physical demanding occupations (^b^) or both (^c^)

#### Sales (5 studies)

Out of more than 19,500 men working in sales, 93 were diagnosed with HOA. Among these men, the summary of all studies stated an elevated risk by 50% (RR 1.50; 95%-CI 1.01 to 2.24) with moderate heterogeneity between results (I^2^ = 38%). A subgroup analysis of the results of four of these studies with comparable physically demanding occupations (warehousemen, clerks, service and shop worker) stated this exposure effect, but decreased precision due to decreasing sample size (supplementary table S13, Table 2).

#### Gastronomy (4 studies)

Out of 22,716 men working in gastronomy, 82 were diagnosed with HOA. Different studies summarized different occupational groups, which might explain the substantial heterogeneity of results (I^2^ = 63%). Due to this, it was not reasonable to pool the results. Two studies stated an increased risk [34, 36] (supplementary table S14, Table 2).

#### Food production (4 studies)

Out of more than 6,461 workers in food production, 66 were diagnosed with HOA. The results of all studies stated an elevated risk (RR 2.13; 95%-CI 1.53 to 2.95) with small heterogeneity between results (I^2^ = 0%) (Table 2).

#### Storage and transportation worker (3 studies)

Out of more than 3,100 men working as postmen, 22 were diagnosed with HOA. Summarising the results of all studies, we stated a non-significantly elevated risk increase (RR 2.09; 95%-CI 0.88 to 4.96) with small heterogeneity between results (I^2^ = 0%) (Table 2).

#### Health care (3 studies)

Out of 99,603 men working in health care, 321 were diagnosed with HOA. Exposures and diagnostic criteria were very heterogeneous and explain substantial heterogeneity of results (I^2^ = 88%) (supplementary table S17, Table 2).

#### Unskilled or basic level labour (3 studies)

Out of 19,273 men working in unskilled or basic level labour, 383 were diagnosed with HOA. All studies stated an elevated risk with an increase of 89% (RR 1.89; 95%-CI 1.29 to 2.77) compared to jobs requiring higher qualification with moderate heterogeneity between results (I^2^ = 34%) (Table 2).

In subgroup analyses, we investigated possible sources of heterogeneity and stated no difference of the results depending on different diagnostic criteria and study types (Table 2).

## Discussion

This systematic review reveals considerable differences between different physically demanding occupations regarding developing HOA and consequential sick leaves, hospitalisation, THR or disability pensions. We identified six occupational groups with evidence for an increased risk of HOA compared to professionals with no or low physical strain. These groups included workers in agriculture, fishery or forestry, in food production or sales, construction and metal workers and men driving vehicles with whole body vibration.

Our results correspond to the conclusions of recent systematic reviews that heavy physical workload exposure on the job increases the risk of developing HOA [17, 18, 20, 46]. A greater exposure to manual handling of weights at work is associated with a higher risk to develop HOA [47, 48] and can even double this risk [19]. But our results show that occupations demanding various other physical exercises can also increase the risk of HOA. Thus, they are in line with the results from a recent systematic review initiated by the World Health Organisation (WHO) and the International Labour Organisation (ILO) with the aim to develop estimates of work-related burdens to ergonomic risk factors. These factors included force exertion, demanding postures, repetitiveness, vibrations and lifting [49]. Occupations in agriculture, fishery or forestry, construction and metal industry require frequent lifting and carrying of heavy loads and demanding working postures. Food production requires repetitive activities, long standing and running, but workers have to lift and carry of medium loads. Driving vehicles is a predominantly sedentary activity with lack of movement, but with whole body vibrations, which may damage joint cartilage.

For two of these occupational groups with increased risk of HOA, we identified a substantial heterogeneity between results of different studies. These groups will be discussed in detail to explain these different results.

First, we stated the highest risk increase to develop HOA was for men working in agriculture, fishery or forestry with regular long working days, few vacations and different demanding working positions. Due to the large magnitude of the effect and the presence of a dose–response gradient we see evidence of an cause-effect relationship between occupations in agriculture, fishery and forestry and the development of HOA. But despite high prevalence of musculoskeletal disorders [50], the total number of farmers who take time off and seek care [51] is low and it is unclear if farmers, who are very dependent on their mobility, are motivated to accept their situation earlier or to delay THR and continue to work or accept a disability pension. But this evidence is limited by the substantial heterogeneity between the results of different studies. This heterogeneity might be caused by the high variability in the age of farmers, time periods to calculate prevalences, differences in the use of machines to facilitate physically demanding work, daily working time and maximal working time during season [51]. Moreover, some authors propose that work with animal handling might result in an additional immunological stimulation of HOA in contrast to in crop production [52], but other aspects like frequent walking on uneven grounds could also be considered in this context.

Nearly all studies stated an increased risk for HOA by driving vehicles where whole body vibrations may damage cartilage resulting in osteoarthritis. However stated risk increases were heterogeneous. The strength of the vibration transmitted to the skeleton depends on many factors including the vehicle itself, the surface, the sitting posture and whether damping has been built into the seat. In addition, construction machinery and agricultural vehicles cause higher levels of vibration than passenger cars. Our results are in line with the results of a German case–control study [19] which found a relationship between heavily vibrating vehicles and the risk of lumbar disc herniation as well as symptomatic lumbar disc narrowing.

We additionally identified a remarkable risk increase of unskilled and basic level workers compared to workers with higher education (RR 1.89; 95%-CI 1.29 to 2.77). These workers are frequently exposed to lifting heavy loads and carrying out physically demanding repetitive work for long periods due to the specialization of many functions in farming [36] and other jobs. Unskilled workers usually have limited possibilities to adjust their work environment to prevent early sick leave, hospitalization and disability retirement due to HOA. These socioeconomic and resulting health inequality differences have been documented in several studies [53, 54].

The results of this review support the need for advanced preventive measures especially in the affected occupational groups in agriculture, construction and food production. Workplace risk assessments regarding manual handling of (heavy) loads, the job-related need of repetitive body movements and intensive-load working postures like forward trunk inclination and torsion, kneeling, squatting, other manual demanding tasks like pushing and pulling of loads and exposure to vibrations should consider HOA as an adverse health effect. Long lasting, highly intensive, biomechanical or physical workload is associated with a wide range of clinical outcomes, mostly degenerative changes [55]. Therefore, preventive measures are mostly not specific to reducing a single adverse health effect like HOA. Evidence from this review may provide support to offer special preventive strategies and tools to reduce workload, to improve technical equipment and organizational aspects and workers’ knowledge to tackle the demands in these occupations with a high risk to develop HOA. Preventive occupational medicine should include examination of the hip after no more than 15 to 20 years in the identified physically demanding occupations. Early detection and management of HOA might prevent or delay hospital admissions, support return to work and rehabilitation efforts [9], and avoid severe consequences like total hip replacement, disability retirement or occupational disease claims due to HOA.

### Limitations

The precision of our results is reduced by heterogeneity of exposed occupational groups with intensive physical strain and non-exposed reference groups. Development of HOA was measured with very different diagnoses including clinical and radiological criteria, THR and disability pension. Some of these endpoints do not include all cases of HOA. This substantial heterogeneity of exposures and diagnosis of HOA resulted in a low precision with a broad range of results. Finally, BMI as important confounder was not.

Our systematic review is restricted to studies with publications in English and German language. Thus, we might have missed studies published in other languages including Chinese and Russian. Despite these limitations, we were able to identify a number of occupations with an increased risk to develop HOA.

## Conclusion

Occupations with high physical workload to manually handle heavy loads, with intensive-load working demanding postures, repetitive activities or exposures to vibrations increase the risk of HOA with resulting pain, sick leave, hospital admissions, THR and disability retirement. Evidence from this review may support the discussion to define HOA as an occupational disease and to develop special preventive strategies and technical equipment to reduce the impact of mechanical occupational exposure at work sites with high physical strain .

## Supplementary Information

**Additional file 1: Table S1.** Search strategy in Medline (Ovid). **Table S2.** Search strategy in Embase. **Table S3.** Search strategy in the Cochrane Library. **Table S4.** Search strategy in CINAHL. **Table S5.** Search strategy in HSE-Line. **Table S6.** Search in Occupational medicine journals 

**Additional file 2:** **Table S7.** Characteristics of eligible cohort studies. **Table S8.** Characteristics of eligible case-control studies. **Table S9.** Summary of reported results on the association of occupations in agriculture, fishery or forestry and the risk to develop hip osteoarthritis (HOA). **Table S10.** Summary of reported results on the association of occupations in construction and the risk to develop hip osteoarthritis (HOA). **Table S11.** Summary of reported results on the association of occupations with whole body vibrations and the risk to develop hip osteoarthritis (HOA). **Table S12.** Summary of reported results on the association of occupations in metal work and the risk to develop hip osteoarthritis (HOA). **Table S13.** Summary of reported results on the association of occupations in sales and the risk to develop hip osteoarthritis (HOA). **Table S14.** Summary of reported results on the association of occupations in gastronomy and the risk to develop hip osteoarthritis (HOA). **Table S15.** Summary of reported results on the association of occupations in food production and the risk to develop hip osteoarthritis (HOA). **Table S16.** Summary of reported results on the association of occupations in storage and transportation and the risk to develop hip osteoarthritis (HOA). **Table S17.** Summary of reported results on the association of occupations in health care and the risk to develop hip osteoarthritis (HOA). **Table S18.** Summary of reported results on the association of unskilled and basic level labour and the risk to develop hip osteoarthritis (HOA).

## Data Availability

All data generated or analysed during this study are included in this published article.

## Abbreviations

CI: Confidence Interval
HOA: Hip osteoarthitis
RR: Relative risk
THR: Total hip replacement
