# Supplementary material for: Influence of physically demanding occupations on the development of osteoarthritis of the hip: a systematic review
Source: J Occup Med Toxicol. 2022 Aug 24;17:18. doi: 10.1186/s12995-022-00358-y (PMC9400208; doi:10.1186/s12995-022-00358-y)
Supplement: Supplementary file 1 — Additional file 1: Table S1. Search strategy in Medline (Ovid). Table S2. Search strategy in Embase. Table S3. Search strategy in the Cochrane Library. Table S4. Search strategy in CINAHL. Table S5. Search strategy in HSE-Line. Table S6. Search in Occupational medicine journals [file 12995_2022_358_MOESM1_ESM.docx]

# Search strategies

## Medline (Ovid)

Table S1: Search strategy in Medline (Ovid)

| **Search terms** (24.02.2016/ 18.06.2018 / 04.05.2020) |
| --- |
| **Exposure: Occupational physical activities and groups** |
| 1 exp Occupational disease/ |
| 2 exp Occupational health/ |
| 3 exp Occupational Exposure/ or occupational exposure.mp. |
| 4 exp Occupational Medicine/ |
| 5 Work-related.mp. |
| 6 Working environment.mp. OR work environment.mp. |
| 7 at work.mp. |
| 8 exp occupations/ OR Occupation*.mp. |
| 9 exp work/ OR worke*.mp. |
| 10 (workplace or (work place)).mp. |
| 11 (worksite or (work site)).mp. |
| 12 job*.mp. |
| 13 exp occupational groups/ |
| 14 ((work load) or workload or heavy load).mp. |
| 15 exp employment/ |
| 16 exp industry/ |
| 17 exp agriculture/ |
| 18^a^ (lifting or (weight bearing) or weightbearing).mp. |
| 19 (physical* adj1 demand* work*).mp. |
| 20^a^ (whole adj1 body vibrat*).mp. |
| 21^a^ (heavy carrying or (physical strain) or (constrain* postur*) or kneeling or squatting).mp |
| 22 farmer.mp. |
| 23 (construct* work).mp. |
| 24^a^ (health adj1 care work*).mp. |
| 25^a^ (hip adj flex*).mp. |
| **26 or/1-25** |
| **Outcome** |
| 27 exp Osteoarthritis, Hip/ |
| 28 ((osteoarthritis hip) or (hip osteoarthritis)).mp. |
| 29 coxarthros*.mp. |
| 30 (hip adj1 joint).mp. |
| 31 (hip adj3 endoprothes*).mp. |
| 32 (hip adj3 prosthes*).mp. |
| 33 (hip replace*).mp. |
| 34 (hip adj (pain or mobilit* or symptom*)).mp. |
| 35 ((function* disability*) and hip).mp. |
| 36 (motion* measur* and hip).mp. |
| 37 exp Arthroplasty/ |
| **38 or/27-37** |
| 39 26 and 38 |
| 40 exp animals / not humans |
| 41 39 not 40 |
| 42 limit 41 to yr="1990 -Current" |
| 43 limit 42 to (English or German) |
| a: part of the search strategy 2015 on the association between between occupational workload and the development of HOA, not included in the searches 2018 and 2020 |

## Embase

Table S2: Search strategy in Embase

| **Search terms** (12.3.2015) |
| --- |
| 1. exp occupational disease/ |
| 2. exp Occupational health/ |
| 3. occupational exposure.mp. or exp occupational exposure/ |
| 4 exp occupational medicine/ |
| 5 Work-related.mp. |
| 6 Working environment.mp. OR work environment.mp. |
| 7 at work.mp. |
| 8 exp occupations/ OR Occupation*.mp. |
| 9 exp work/ OR worke*.mp. |
| 10 (workplace or (work place)).mp. |
| 11 (worksite or (work site)).mp. |
| 12 job*.mp. |
| 13 exp occupational groups/ |
| 14((work load) or workload or heavy load ).mp. |
| 15 exp employment/ |
| 16 exp industry/ |
| 17 exp agriculture/ |
| 18 (lifting or (weigt bearing) or weigtbearing).mp. |
| 19 (physical* adj1 demand* work*).mp. |
| 20 (whole adj1 body vibrat*).mp. |
| 21 (heavy carrying or (physical strain) or (constrain* postur*) or kneeling or squatting).mp |
| 22 farmer.mp. |
| 23 (construct* work).mp. |
| 24 (health adj1 care work*).mp. |
| 25 (hip adj flex*).mp. |
| **26 or/1-25** |
| 27 exp Osteoarthritis, Hip/ |
| 28 ((osteoarthritis hip) or (hip osteoarthritis)).mp. |
| 29 coxarthros*.mp. Or coxarthrit*.mp. |
| 30 (hip adj1 joint).mp. |
| 31 (hip adj3 endoprothes*).mp. |
| 32 (hip adj3 prosthes*).mp. |
| 33 (hip replace*).mp. |
| \| 34 (hip adj (pain or mobilit* or symptom*)).mp. \| (hip adj (pain or mobilit* or symptom*)).mp. \| \| --- \| --- \| |
| 35 ((function* disability*) and hip).mp. |
| 36 (motion* measur* and hip).mp. |
| 37 exp Arthroplasty/ |
| 38 (osteoartheros*  and hip).mp |
| **39 or/27-38** |
| 40 26 and 39 |
| 41 exp animals / not humans |
| 42 40 not 41 |
| **43 limit 42 to yr="1990 -Current"** |

## Cochrane Library

*Table S3: Search strategy in the Cochrane Library*

| **Search terms** (24.2.2015/(18.06.2018 / 04.05.2020) |
| --- |
| #1 MESH descriptor Osteoarthritis, Hip explode all trees |
| #2 hip near osteoarthritis |
| #3 coxarthros* |
| #4 hip near (joint or endoprothes* or prosthes* or replace*) |
| #5 hip near (pain or mobilit* or symptom*) |
| #6 hip and (function* disabilit*) |
| #7 hip and (Motion* measur*) |
| #8 MESH descriptor Arthroplasty, Replacement explode all trees |
| #9 #1 or #2 or #3 or #4 or #5 or #6 or #7 or #8 |
| #10 MESH descriptor Occupational Diseases explode all trees |
| #11 MESH descriptor Occupational Health explode all trees |
| #12 MESH descriptor Occupational Exposure explode all trees |
| #13 MESH descriptor Occupational Medicine explode all trees |
| #14 work near (related or Environment or place or site or load) |
| #15 at work |
| # 16 MESH descriptor occupations explode all trees |
| # 17 occupation* Job* |
| # 18 MESH descriptor work explode all trees |
| # 19 MESH descriptor Occupational Groups explode all trees |
| # 20 Job* |
| # 21 MESH descriptor employment explode all trees |
| # 22 MESH descriptor industry explode all trees |
| # 23 MESH descriptor agriculture explode all trees |
| #24 lift* or carry* |
| # 25 physical* near (demand* work*) |
| # 26^a^ whole near (body vibrat*) |
| # 27 farmer* |
| # 28 construct* near work* |
| # 29 health care near work* |
| # 30^a^ hip near flex* |
| # 31 #10 or #11 or #12 or #13 or #14 or #15 or #16 or #17 or #18 or #19 or #20 or #21 or #22 or #23 or #24 or #25 or #26 or #27 or #28 or #29 or #30 |
| #32 #31 AND #9 |
| a: part of the search strategy 2015 on the association between between occupational workload and the development of HOA, not included in the searches 2018 and 2020 |

## CINAHL

Table S4: Search strategy in CINAHL

Search terms (4.05.2020):

osteoarthritis, hip or coxarthrosis or hip replacement or hip endoprosthesis in Abstract

AND

occupation or job or career or profession or employment or industry or agriculture or farmer or construction or health care in Titel

## HSE-Line

Table S5: Search strategy in HSE-Line

| **Nr.** | **Search terms** (03.03.2015/ 06.05.2020)  **(**http://www.hse.gov.uk/infoserv/hseline.htm**)** |
| --- | --- |
| 1 | osteoarthritis and hip |
| 2 | *Coxarthros* |
| 3 | Hip and endoprosthesis |
| 4 | Hip and replacement |

## [Occupational](file:///C:\Users\unversu\AppData\Local\Microsoft\Windows\INetCache\Content.Outlook\01%20Aktuelle%20Suche\Suchabfrage\Handsuche%20Journals%20exportierte%20Artikel) medicine journals

Table S6: Search in occupational medicine journals

| **Journal (May 2020)** |
| --- |
| Occupational medicine (Oxford, England) |
| The journal of the Society of Occupational med. |
| Occupational medicine (Philadelphia, Pa.) |
| Clinics in occupational and environmental med. |
| International journal of occupational and environmental med. |
| Journal of occupational medicine |
| Internat. journal of occup. Med. and environmental health |
| Polish journal of occup. Med. and environmental health |
| Journal of occupational med. and toxicology (London, Engl.) |
| Journal of occupational health |
| Journal of environmental and occupational science |
| Scandinavian Journal of work, environment & health |
| Safety and health at work |
| Occupational and environmental medicine |
| British journal of industrial medicine |
| Archives of environmental & occupational health |
| International journal of occup. and environmental health |
