# Supplementary material for: Influence of physically demanding occupations on the development of osteoarthritis of the hip: a systematic review
Source: J Occup Med Toxicol. 2022 Aug 24;17:18. doi: 10.1186/s12995-022-00358-y (PMC9400208; doi:10.1186/s12995-022-00358-y)
Supplement: Supplementary file 2 — Additional file 2: Table S7. Characteristics of eligible cohort studies. Table S8. Characteristics of eligible case-control studies. Table S9. Summary of reported results on the association of occupations in agriculture, fishery or forestry and the risk to develop hip osteoarthritis (HOA). Table S10. Summary of reported results on the association of occupations in construction and the risk to develop hip osteoarthritis (HOA). Table S11. Summary of reported results on the association of occupations with whole body vibrations and the risk to develop hip osteoarthritis (HOA). Table S12. Summary of reported results on the association of occupations in metal work and the risk to develop hip osteoarthritis (HOA). Table S13. Summary of reported results on the association of occupations in sales and the risk to develop hip osteoarthritis (HOA). Table S14. Summary of reported results on the association of occupations in gastronomy and the risk to develop hip osteoarthritis (HOA). Table S15. Summary of reported results on the association of occupations in food production and the risk to develop hip osteoarthritis (HOA). Table S16. Summary of reported results on the association of occupations in storage and transportation and the risk to develop hip osteoarthritis (HOA). Table S17. Summary of reported results on the association of occupations in health care and the risk to develop hip osteoarthritis (HOA). Table S18. Summary of reported results on the association of unskilled and basic level labour and the risk to develop hip osteoarthritis (HOA). [file 12995_2022_358_MOESM2_ESM.docx]

# Additional file 3. Supplementary Tables

## Study characteristics

### Cohort studies

*Table S7: Characteristics of eligible cohort studies*

| **Study**  Country | **Population**  inclusion criteria, N, age, gender (% men) at follow-up | **Exposition (occupational groups)**  **vs. Reference**  Source, period | **Outcome (HOA)**  Diagnosis (time)  Follow-up | **Quality score**  Total (S/E/C/D) |
| --- | --- | --- | --- | --- |
| **Andersen 2012**  Denmark | Population-based register data of 5 occupational groups, ≥ 16 years (1981-2006)  N=2 117 298  Age (median): 36-44 years in the groups Men: 18.1 – 97.8 % in the groups | Register data of the income register in the basis on work skills/position and education (1981-2006)  Floor layers/bricklayers/pavers, construction workers, farmers, healthcare assistants) vs.  office workers | Surgically treated HOA (ICD, 1996 to 2006)  Follow-up: 8.1-9.2 years | **15** (4/3/3/5) |
| **Hubertsson 2017**  Sweden | Population-based register data from a region of included occupational groups, 40-70 years (2008-2012)  N=165 179  Age (mean): 55 years  Men: 41.7 % | Register data with classifications of current jobs (12/2012)  7 occupational groups at a higher risk of HOA including more female-dominated occupations vs.  business and administration (e.g. business and finance professionals, mathematicians, statisticians, computing professionals, secretaries and numerical clerks) | Sick leave and disability pension due to HOA (ICD, 2007-2012)  Follow-up: ≤ 6 years | **12** (4/3/2/3) |
| **Järvholm 2004**  Sweden | Cohort of male construction worker participating in a national health control program, BMI 17-35, 15-67 years (1960-1992)  N=204 741  Age (range): 40-79 (33), 20-84 (34) years  Men: 100 % | occupational title at the time of health check (1960-1992)  20 occupations in construction work vs.  white collar worker (33)  5 groups of exposure to whole body vibration vs.  office workers (34) | Surgically treated HOA (ICD, register code for THR, 1987-1998)  Follow up: 3-28 years | **12** (3/2/4/3) |
| **Solovieva 2018**  Finland | Population-based register data from a 70 % random sample of persons with gainful jobs, 30-60 years (01/2005)  N= 1 135 654  Age (mean): 42-48 years in occupational groups  Men: 50.6 % | Register data of longitudinal employer-employee data with classifications (12/2004)  18 occupational groups vs.  professionals (e.g. physical, mathematical, and engineering science professionals,  life science and health professionals, as well as others ) | Full-time disability retirement due to HOA (ICD, 2005-2013)  Follow-up: 9 years | **14** (4/2/4/4) |
| **Thelin 2007**  Sweden | Birth register cohort of male farmers (1930-1949) and a rural reference population of occupational active men (1989)  N= 1220 farmer at baseline  Age: 40-59 years  Men: 100 % | Farmer (≥ 25 hours per week in agriculture) identified from a national farm register vs.  urban references (matched to farmers by age and residential area)) | HOA or surgery of the hip (ICD, 1997-2003)  Follow-up: ≥13 years | **15** (4/2/3/6) |
| **Tuechsen 2003**  Denmark | 4 consecutive cohorts of all gainfully employed Danish men (01/ 1981, 1986, 1991, 1994)  N: n.d.  Age (range): 20-59 years  Men: 100 % | Most important occupation (1980, 1985, 1990, 1993) from register data  observed number vs. expected number of cases for 17 occupational groups with statistically significant relative risks are reported | First hospital admission due to HOA (ICD, 1981-85, 1981-1985, 1986-90, 1991-93, 1994-99)  Follow-up: 3-6 years | **12** (5/3/3/1) |
| **Vingard 1991**  Sweden | Data from register sampling of persons, born 1905-1945 with the same occupation in 1960 and 1970 (1980)  N: 250 217  Age (range): 35-75 years  Men: 83 %* | Blue-collar occupations with high (more than average) (14 for males, 5 for females) vs.  low (less than average) exposure to dynamic or static forces acting on lower extremity | Hospital care for HOA (ICD, 1981-83)  Follow-up ≥ 15 years | 13 (4,2,2,5) |
| C: comparability; D: diagnosis; E: exposure; HOA: Hip osteoarthritis; ICD: International classification of diseases (version 8-10); n.d.: no data; S: selection; SES: socio-economic status; THR: total hip replacement | | | | |

### Case-control studies

*Table S8: Characteristics of eligible case-control studies*

| **Study**  Country | **Population**  inclusion criteria, N (cases/controls), response, age, gender (% male) | **Description Cases vs. Controls**  diagnostic criteria, period | **Exposition (occupational group) vs. reference**  period | **Quality score**  Total (S/A/E) |
| --- | --- | --- | --- | --- |
| **Croft 1992a**  UK | All outpatients with i.v. urograms (1982-87)  N=787 (353 /434)  Response: 69% / 68%  Age (range): 60-75 years Men: 100 % | **cases:** THR for HOA or a joint space ≤ 2.5 mm or ≤ 1.5 mm in ≥ 1 hip  **controls:** joint space ≥ 3.5 mm in both hips and no other radiographic evidence on HOA  1982-1987 | **Interview** on occupational history, coded into 8 groups with time periods for farmer vs. no farmer | **9** (2/3/4) |
| **Croft 1992b**  UK | Random sample of outpatients from rural general practices,  N=250 (30/220)  response: 72 % of contacted, 51 % of eligible were included  Age (range): 60-76 years  Men: 100 % | THR confirmed by hospital, radiograph from the past 6 months or invitation for radiography  **cases:** THR for HOA or a joint space ≤ 1.5 mm in ≥ 1 hip  **controls:** all other participants  1982-1987 | Screening postal **questionnaire** and structured interview on occupational history in farming (years and type) vs. office worker | **9** (2/3/4) |
| **Elsner 1995**  Germany | Patients of an orthopaedic practice  N=418 (220/198)  Response cases: 60 %, control: n.d.  Age (range) of cases: 30 % - > 60 years with younger controls  Men: 61 % | **cases:** patients with hip symptoms and radiographic signs of HOA  **controls:** persons without hip symptoms from a general practice and an ophthalmologist, church community  1989-1993 | **Questionnaire** on occupational history, occupational groups named by ≥ 5 persons vs. all other men or women | **5** (1/2/2) |
| **Franklin 2010**  Iceland | All patients and their first-degree relatives ≥ 60 years  Response cases: 33 %  N= 2490 (1408/1082)  Age: cases: 71-75/ controls: 71 years  Men: 43 % | **cases:** all patients with TKR or THR due to OA, at surgery (1967-1998)  **controls:** first-degree relatives  1998 | **Questionnaire** on occupational history, longest held occupations coded into 8 groups,  6 groups vs. manager and professionals (e.g. teachers, doctors, nurses) | **10** (3/4/3) |
| **Thelin 1997**  (  Sweden | All patients with radiographs of the pelvis and hip joint of 3 departments and controls from the local population register  N=807 (269/538)  Response: 86 (80/89) %  Age (range): < 70 years  Men: 100 % | **cases:** radiological verified HOA with joint space < 3 mm ≥ 1 hip  **controls:** matched by age, gender, place of residence  1986-88, re-evaluated 1989 | **Questionnaire** on occupational history, coded into 7 main group, comparisons to all other men and farmer (with time period) vs. no farmer | **10** (4/3/3) |
| **Vingard 1992**  Sweden | all men from Stockholm county born in 1915 to 1934 (register)  N=438 (140/298)  Response: n.d.  Age (range): 46-65 Jahre  Men: 100 % | **cases:** disability pension by hip OA, knee OA, low back disorders, neck or shoulder  **controls**: random sample in the same age group  1979,1980,1981,1984 | **Interviews** on occupational history until 1981,  only analyses of 20 most exposed occupations (≥ 10 years ) vs. all men never exposed to any in these are reported | **10** (4/2/4) |
| A: adjustment; E: exposition; HOA: Hip osteoarthritis; n.d.: no data; S: selection; THR: total hip replacement | | | | |

## Agriculture, fishery and forestry

*Table S9: Summary of reported results on the association of occupations in agriculture, fishery or forestry and the risk to develop hip osteoarthritis (HOA)*

| **Study** | **Occupational subgroups** | **Outcome** | **N** | **n (%), IR per 100.000 PY (95%CI)** | **Effect (95 %-CI95 %-CI)** |
| --- | --- | --- | --- | --- | --- |
| **Andersen** **2012** (CS) | | | | | |
| Exposure | Farmer | HOA | 161 923^a^ | 3361 (2,1%)^a^,  IR: 157,7^b^ | HR: 1.96 (1.84; 2.08)*^b^ |
| Reference | Office worker |  | 316 543^a^ | 2475 (0,8%)^a^,  IR: 60,2^b^ |  |
| **Croft 1992b** (CCS) | | | | | |
| Exposure | Farmer (≥ 10 years) | HOA | 115 | 22 (19.1 %)^a^ | OR: 9.3 (1.9; 44.5)*^c^ |
| Exposure | Farmer (1-9 years) |  | 52 | 6 (3.9 %)^a^ | OR: 4.5 (0.8; 26.3)^c^ |
| Reference | Men with mainly sedentary jobs |  | 83 | 2 (2.4 %)^a^ |  |
| **Croft 1992a** (CCS) | | | | | |
| Exposure | Farmer and agricultural worker | THR for HOA or a joint space ≤ 2.5 mm | 117^a^ | 52 (44.4 %)^a^ | OR: 0.9 (0.6; 1.4)^b^ |
| Reference | All other men |  | 422^a^ | 193 (45.7 %)^a^ |  |
| Exposure | Farmer and agricultural workers | THR for HOA or a minimal joint space ≤ 1.5 mm | 117^a^ | 19 (16.2 %)^a^ | OR: 1.6 (0.8; 3.1)*^b^ |
| Reference | All other men |  | 422^a^ | 226 (53.6 %)^a^ |  |
| **Elsner 1995** (CCS) | | | | | |
| Exposure | Farmer and forest workers | HOA | 10 | 7 (70.0 %)^a^ | OR: 1.4 (0.31; 6.79)*^b^ |
| Reference | All other men |  | 219 | 127 (58.0 %)^a^ |  |
| **Franklin 2010** (CCS) | | | | | |
| Exposure | Farmer | THR | n.d. | 140 [hip]  10 [hip and knee] | OR: 3.6 (2.1; 6.2)*^b^ |
| Reference | Manager and professionals |  | n.d. | 27 [hip]  3 [hip and knee] |  |
| **Hubertsson 2017** (CS) | | | | | |
| Exposure | Farmer (market gardeners, crop growers, marked-oriented animal producers) | Sick leave due to HOA | 5123 | 35 (0.68 %)^a^ | OR: 1.56 (1.03; 2.38)^c^ |
| Reference | Business and administration |  | 22 556 | 76 (0.34 %)^a^ |  |
| Exposure | Farmer | Disability pension due to HOA | 5123 | 10 (0.20 %)^a^ | OR: 3.15 (1.16; 8.55)*^c^ |
| Reference | Business and administration |  | 22 556 | 7 (0.03 %)^a^ |  |
| **Solovieva 2018** (CS) | | | | | |
| Exposure | Agricultural and fishery worker | Full disability retirement due to HOA | 34 477^a^ | 135 (0.39 %)^a^, IR: 49 (35; 70)^f^ | HR: 1.92 (0.95; 3.89)*^c^ |
| Reference | Professionals |  | 77 573^a^ | 26 (0.034 %)^a^, IR: 4 (2; 8)^b^ |  |
| **Thelin 1997** (CCS) | | | | | |
| Exposure | Farmer | Radiological confirmed HOA | 321 | 136 (42.4 %) | OR: 2.70 (1.94; 3.77)*^b^ |
| Reference | Never worked in farming |  | 374 | 80 (21.4 %) |  |
| Exposure | Farming as farmer (1-10 years) |  | 20 | 6 (30.5 %) | OR: 1.58 (0.59; 4.23)^b^ |
| Exposure | Farming as farmer (11-20 years) |  | 30 | 13 (43.3 %) | OR: 2.81 (1.31; 6.03)^b^ |
| Exposure | Farming as farmer (21-30 years) |  | 21 | 14 (66.7 %) | OR: 7.35 (2.87; 18.82)^b^ |
| Exposure | Farming as farmer (> 30 years) |  | 102 | 52(51.0 %) | OR: 3.82 (2.41; 6.06)^b^ |
| Reference | Never worked in farming |  | 374 | 80 |  |
| **Thelin 2007** (CS) | | | | | |
| Exposure | Farmer | Low back symptoms | 657 | 445^a^ (67.7 %) | OR: 1.51 (1.02; 2.23)^b^ |
| Reference | Non-farmer (matched) |  | 657 | 379^a^ (57.7 %) |  |
| Exposure | Farmer | Hip symptoms | 657 | 208^a^ (31.7 %) | OR: 1.46 (1.11; 1.93)^b^ |
| Reference | Non-farmer (matched) |  | 657 | 142^a^ (21.6 %) |  |
| Exposure | Farmer | HOA | 1220 | 51 (4.2 %) | HR. 3.0 (1.7; 5.3)*^b^ |
| Reference | Urban references (matched) |  | 1220 | 15 (1.4 %) |  |
| **Tüchsen 2003** (CS) | | | | | |
| Exposure | Self-employed farmer, period 1994-99 (last reported period) | Hospitalisation due to THR due to HOA | 34 068 | 355 (1.0 %)^a^ | SHR: 2.86 (2.62;3.13)*^b^ |
| Exposure | Employee in agriculture and horticulture, period 1994-99 (last reported period) |  | 20 734 | 97 (0.47 %)^a^ | SHR: 1.89 (1.58;2.27)^b^ |
| Reference | All gainfully employed men |  | n.d. | n.d. | 1 |
| **Vingard 1991** (CS) | | | | | |
| Exposure | Farmer | Hospitalization due to HOA | 35 981 | 479 (1.3 %)^a^ | RR: 3.78 (2.91; 3.88)*^b^ |
| Exposure | Forest worker and log driver |  | 7270 | 35 (0.48 %)^a^ | RR: 1.37 (0.93; 1.87)^b^ |
| Reference | Low exposure blue-collar workers |  | 91 057 | 320 (0.35 %)^a^ |  |
| **Vingard 1992** (CCS) | | | | | |
| Exposure | Farmer and forest worker (≥ 10 years) | Disability pension due to HOA |  | 17 | RR: 13.8 (4.0; 48.1)*^b^ |
| Reference | Low-exposure persons |  | n.d. | n.d. |  |
| Exposure | Farmer, forest worker (≥ 10 years) | Disability pension due to low back disorders |  | 47 | RR: 5.6 (1.8; 18.6)^b^ |
| Reference | Low-exposure persons |  | n.d. | n.d. |  |
| *best correspondence to describe the association between occupations as farmers and HOA  a: calculated by the authors  b: adjusted for age (1 quality point)  c: adjusted or matched for age and other important confounder (2 quality points)  CCS: case-control study; CI: confidence interval; CS: cohort study; HOA: Hip osteoarthritis; HR: Hazard ratio; IR: incidence rate; N: number of persons; n.d.: no data; n: number of cases; OR: odds ratio; PY: person years; QS: quality score; RR: relative risk; SHR: standardized hospitalization ratio ((observed / expected number); THR: hip replacement therapy | | | | | |

## Construction worker

*Table S10:*  *Summary of reported results on the association of occupations in construction and the risk to develop hip osteoarthritis (HOA)*

| **Study** | **Occupational subgroups** | **Outcome** | **N** | **n (%), IR (95%CI) per 100.000 PY** | **Effect (95 %-CI95 %-CI)** |
| --- | --- | --- | --- | --- | --- |
| **Andersen 2012** (CS) | | | | | |
| Exposure | Construction worker | HOA | 448 671^a^ | 2899 (0.6 %)^a^,  IR: 89.9^b^ | HR: 1.23 (1.15; 1.31)*^b^ |
| Exposure | Floor- and bricklayer |  | 4285^a^ | 326 (7.6 %)^a^,  IR: 99.5^b^ | HR: 1.35 (1.20; 1.52)^b^ |
| Reference | Office worker |  | 316 543 | 1473 (0.5 %)^a^,  IR: 89.6^b^ |  |
| **Croft 1992a** (CCS) | | | | | |
| Exposure | Construction worker and labourers | THR for HOA or a joint space ≤ 2.5 mm | 72^a^ | 35 (48.6 %)^a^ | OR: 1.2 (0.7; 1.9)^b^ |
| Reference | All other men |  | 467 ^a^ | 210 ^a^ (45.0 %)^a^ |  |
| Exposure | Construction worker and labourers | THR for HOA or a minimal joint space ≤ 1.5 mm | 46^a^ | 9 (19.6 %)^a^ | OR: 1.5 (0.7; 3.4)*^b^ |
| Reference | All other men |  | 493 ^a^ | 236 ^a^ (47.9 %)^a^ |  |
| **Elsner 1995** (CCS) | | | | | |
| Exposure | construction workers | HOA | 33 | 22 (66.7 %)^a^ | OR: 1.1 (0.53; 2.57)*^b^ |
| Reference | All other men |  | 196 | 112 (57.1 %)^a^ |  |
| **Franklin 2010** (CCS) | | | | | |
| Exposure | Craft worker (as carpenter, construction worker, fish processing and sewer) | THR | n.d. | 78 [hip]  6 [hip and knee] | OR: 1.5 (0.87; 2.7)*^b^ |
| Reference | Manager and professionals |  | n.d. | 27 [hip]  3 [hip and knee] |  |
| **Hubertsson 2017** (CS) | | | | | |
| Exposure | Construction (civil engineers, civil engineering technicians) | Sick leave due to HOA | 20 162 | 114 (0.57 %)^a^ | OR: 1.46 (1.06; 2.00)^c^ |
| Reference | Business and administration, |  | 22 556 | 76 (0.34 %)^a^ |  |
| Exposure | Construction | Disability pension due to HOA | 20 162 | 23 (0.11 %)^a^ | OR: 2.18 (0.90; 5.27)*^c^ |
| Reference | Business and administration |  | 22 556 | 7 (0.03 %)^a^ |  |
| **Järvholm 2004** (CS) | | | | | |
| Exposure | Asphalt worker | THR | 2850 | 21 (0.74 %)^a^,  IR: 83.5 (45.8; 121.2)^c^ | RR: 1.50 (0.90; 2.49)^c^ |
| Exposure | Brick layer |  | 7076 | 58 (0.82 %)^a^,  IR: 65.8 (48.6; 83.0)^c^ | RR: 1.16 (0.80; 1.67)^c^ |
| Exposure | Concrete worker |  | 26 178 | 247 (0.94 %)^a^,  IR: 73.0 (63.6; 82.4)^c^ | RR: 1.27 (0.95; 1.70)*^c^ |
| Exposure | Floor layer |  | 3240 | 19 (0.59 %)^a^,  IR: 82.9 (43.9; 121.9)^c^ | RR: 1.58 (0.93; 2.68)^c^ |
| Exposure | Plumber |  | 16 317 | 94 (0.58 %)^a^,  IR: 64.7 (51.5; 77.9)^c^ | RR: 1.15 (0.82; 1.60)^c^ |
| Exposure | Rock worker |  | 2695 | 27 (1.0 %)^a^,  IR: 85.7 (52.3; 119.1)^c^ | RR: 1.37 (0.87; 2.13)^c^ |
| Reference | White collar worker |  | 9136 | 56 (0.61 %)^a^,  IR: 57.5 (42.3; 72.6)^c^ | 1 |
| **Solovieva 2018** (CS) | | | | | |
| Exposure | Construction worker, electricians and plumbers | Full disability retirement due to HOA | 47 693^a^ | 208 (0.44 %)^a^,  IR: 56 (43; 75)^b^ | HR: 1.70 (0.75; 3.84)*^c^ |
| Reference | Professionals |  | 77 573^a^ | 26 (0.034 %)^a^,  IR: 4 (2; 8)^b^ |  |
| **Vingard 1991** (CS) | | | | | |
| Exposure | Construction worker | Hospitalization due to HOA | 38 095 | 223 (0.59 %)^a^ | RR: 1.66 (1.32; 1.87)*^b^ |
| Reference | Low exposure blue-collar worker |  | 91 057 | 320 (0.35 %)^a^ |  |
| **Vingard 1992** (CCS) | | | | | |
| Exposure | Construction workers | Disability pension due to HOA | n.d. | 27 | RR: 5.3 (2.6; 10.6)*^b^ |
| Reference | Low-exposure persons |  | n.d. | n.d. |  |
| Exposure | Construction worker | Disability pension due to low back disorders | n.d. | 154 | RR: 4.8 (2.7; 8.5)^b^ |
| Reference | Low-exposure persons |  | n.d. | n.d. |  |
| *best correspondence to describe the association between occupations in construction work and HOA  a: calculated by the authors  b: adjusted for age (1 quality point)  c: adjusted or matched for age and other important confounder (2 quality points)  CCS: case-control study; CI: confidence interval; CS: cohort study; HOA: Hip osteoarthritis; HR: Hazard ratio; IR: incidence rate; N: number of persons; n.d.: no data; n: number of cases; OR: odds ratio; PY: person years; QS: quality score; RR: relative risk; THR: hip replacement therapy | | | | | |

## Driving vehicles with whole-body vibration

*Table S11: Summary of reported results on the association of occupations with whole body vibrations and the risk to develop hip osteoarthritis (HOA)*

| **Study** | **Occupational subgroups** | **Outcome** | **N** | **n (%), IR pro 100.000 PY (95%CI)** | **Effect (95 %-CI)** |
| --- | --- | --- | --- | --- | --- |
| **Croft 1992a** (CCS) | | | | | |
| Exposure | Truck, bus and car driver (≥ 1 year) | THR for HOA or a joint space ≤ 2.5 mm | 92^a^ | 41 (44.6 %)^a^ | OR: 0.9 (0.6; 1.4)^b^ |
| Reference | All other men |  | 447 ^a^ | 204 (45.6 %)^a^ |  |
| Exposure | Truck, bus and car driver (≥ 1 year) | THR for HOA or a joint space ≤ 1.5 mm | 92^a^ | 11(12.0 %)^a^ | OR: 1.1 (0.5; 2.4)*^b^ |
| Reference | All other men |  | 447 ^a^ | 234 (52.3 %)^a^ |  |
| **Elsner 1995** (CCS) | | | | | |
| Exposure | Any occupation with whole body vibration | HOA | 51 | 37 (72.5 %)^a^ | OR: 2.0 (1.04; 4.20)*^b^ |
| Reference | All other men |  | 177 | 97 (54.8 %)^a^ |  |
| Exposure | Any occupation with partial body vibration | HOA | 32 | 24 (75.0 %)^a^ | OR: 2.2 (1.00; 4.99)^b^ |
| Reference | All other men |  | 197 | 110 (55.8 %)^a^ |  |
| Exposure | Car driver | HOA | 33 | 22 (66,6 %)^a^ | OR: 1.5 (0.68; 3.50)^b^ |
| Reference | All other men |  | 196 | 112 (57,1 %)^a^ |  |
| **Hubertsson 2017** (CS) | | | | | |
| Exposure | Motor-vehicle drivers: taxi, bus and lorry drivers | Sick leave due to HOA | 10 049 | 47 (0.47 %)^a^ | OR: 1.17 (0.79-1.72)^c^ |
| Reference | Business and administration |  | 22 556 | 76 (0.34 %)^a^ |  |
| Exposure | Motor-vehicle drivers: taxi, bus and lorry drivers | Disability pension due to HOA | 10 049 | 7 (0.07 %)^a^ | OR: 1.23 (0.41; 3.65)*^c^ |
| **Järvholm 2004** (CS) | | | | | |
| Exposure | Driving vehicles with whole body vibrations (paving machine operators, earth movers, tractor and loading machine operators, dumper driver, roller operator, road grader) | Surgically treated HOA | 5643 | 22 (0.39 %)^a^,  IR: 35 | RR: 0.82 (0.51; 1.24)*^c^ |
| Reference | Office worker |  | 9855 | 59 (0.60 %)^a^,  IR: 54 |  |
| **Solovieva 2018** (CS) | | | | | |
| Exposure | Professional driver | Full disability retirement due to HOA | 45 969^a^ | 141 (0.31 %),  IR: 39 (28; 56)^i^ | HR: 3.56 (2.15; 5.91)*^c^ |
| Exposure | Machine operators and assemblers |  | 26 432^a^ | 54 (0.20 %),  IR: 26 (15; 46)^i^ | HR: 2.34 (1.32; 4.15)^c^ |
| Reference | Professionals |  | 77 573^a^ | 26 (0.034 %)^a^,  IR: 4 (2; 8)^b^ | 1 |
| **Thelin 1997** (CCS) | | | | | |
| Exposure | Driving | Radiological confirmed HOA | n.d. | 20 | OR: 0.66 (0.39; 1.12)^b^ |
| Exposure | Transportation as current profession or last work before retirement |  | n.d. | 7 | OR: 0.36 (0.16; 0.81)^b^ |
| Exposure | Tractor in agriculture |  | n.d. | 86 | OR: 2.05 (1.45; 2.88)*^b^ |
| Exposure | Forestry machine |  | n.d. | 8 | OR: 1.11 (0.47; 2.64)^b^ |
| Exposure | Dumper (Kipper) |  | n.d. | 1 | OR: 0.27 (0.03; 2.20)^b^ |
| Exposure | Fork-lift |  | n.d. | 8 | OR: 0.47 (0.22; 1.04)^b^ |
| Exposure | Mechanical excavator |  | n.d. | 2 | OR: 0.25 (0.06; 1.11)^b^ |
| Exposure | Tractor in industry |  | n.d. | 15 | OR: 0.95 (0.51; 1.77)^b^ |
| Exposure | Truck |  | n.d. | 29 | OR: 0.72 (0.46; 1.13)^b^ |
| Exposure | Bus |  | n.d. | 5 | OR: 0.92 ( 0.32; 2.65)^b^ |
| Reference | All other men |  | n.d. |  |  |
| **Tüchsen 2003** (CS) | | | | | |
| Exposure | Heavy truck and lorry drivers | Hospitalisation due to THR due to HOA | 25 879 | 82 (0.32 %)^a^ | SHR: 1.36 (1.08; 1.69)*^b^ |
| Exposure | Meat and fish processing machine operators |  | 13 001 | 30 (0.23 %)^a^ | SHR: 1.83 (1.24; 2.62)^b^ |
| Exposure | Wood product machine operator |  | 6940 | 17 (0.24 %)^a^ | SHR: 2.05 (1.19; 3.28)^b^ |
| Exposure | Motor vehicle driver (not elsewhere classified) |  | 9062 | 43 (0.47 %)^a^ | SHR: 1.48 (1.07; 1.99)^b^ |
| Exposure | Machine operators and assemblers (not elsewhere classified) |  | 3367 | 16 (0.48 %)^a^ | SHR: 2.07 (1.18; 3.36)^b^ |
| Exposure | Self-employed people in agricultural tractor pools, period 1994-99 (last reported period) |  | 1157 | 5 (0.4 %)^a^ | SHR: 1.83 (0.86;3.87)^b^ |
| Reference | All gainfully employed men |  | n.d. | n.d. | 1 |
| **Vingard 1991** (CS) | | | | | |
| Exposure | Truck and crane operator | Hospitalisation due to HOA | 5735 | 24 (0.42 %)^a^ | RR: 1.19 (0.79; 1.81)*^b^ |
| Exposure | Forest worker and log driver |  | 7270 | 35 (0.48 %)^a^ | RR: 1.37 (0.93; 1.87)^b^ |
| Reference | Low exposure blue-collar workers |  | 91 057 | 320 (0.35 %)^a^ |  |
| *best correspondence to describe the association between occupations with whole body vibrations due to driving vehicles and HOA  a: calculated by the authors  b: adjusted or matched for age (1 quality point)  c: adjusted or matched for age and other important confounder (2 quality points)  CCS: case-control study; CI: confidence interval; CS: cohort study; HOA: Hip osteoarthritis; HR: Hazard ratio; IR: incidence rate; N: number of persons; n.d.: no data; n: number of cases; OR: odds ratio; PY: person years; QS: quality score; RR: relative risk; SHR: standardized hospitalization ratio (observed / expected number); THR: total hip replacement | | | | | |

## Metal work

*Table S12* *Summary of reported results on the association of occupations in metal work and the risk to develop hip osteoarthritis (HOA)*

| **Study** | **Occupational subgroups** | **Outcome** | **N** | **n (%), IR (95%CI) per 100.000 PY** | **Effect (95 %-CI)** |
| --- | --- | --- | --- | --- | --- |
| **Elsner 1995** (CCS) | | | | | |
| Exposure | Metal occupation | HOA | 42 | 33 (78.6 %)^a^ | OR: 2.5 (1.18; 5.70)*^b^ |
| Reference | All other men |  | 187 | 101 (54.0 %)^a^ |  |
| **Hubertsson 2017** (CS) | | | | | |
| Exposure | Metal work (metal molders, welders, sheet-metal workers, structural-metal preparers and related trades workers) | Sick leave due to HOA | 3088 | 19 (0.62 %)^a^ | OR: 1.60 (0.94; 2.70)^c^ |
| Reference | Business and administration |  | 22 556 | 76 (0.34 %)^a^ |  |
| Exposure | Metal work | Disability pension due to HOA | 3088 | 4 (0.13 %)^a^ | OR: 2.38 (0.67; 8.39)*^c^ |
| Reference | Business and administration |  | 22 556 | 7 (0.03 %)^a^ |  |
| **Järvholm 2004** (CS) | | | | | |
| Exposure | Sheet-metal worker | THR | 6860 | 26 (0.38 %)^a^,  IR: 55.5 (32.7; 78.2)^c^ | RR: 1.10 (0.67; 1.80)*^c^ |
| Reference | White collar worker |  | 9136 | 56 (0.61 %)^a^,  IR: 57.5 (42.3; 72.6)^c^ |  |
| **Solovieva 2018** (CS) | | | | | |
| Exposure | Metal and machinery worker | Full disability retirement due to HOA | 62 059^a^ | 194 (0.31 %)^a^,  IR: 37 (28; 50)^b^ | HR: 1.71 (0.86; 3.40)*^c^ |
| Reference | Professionals |  | 77 573^a^ | 26 (0.034 %)^a^,  IR: 4 (2; 8)^b^ |  |
| Exposure | Chemical, wood and metal processing worker | Full disability retirement due to HOA | 18 962^a^ | 38 (0.20 %)^a^,  IR: 25 (14; 47)^b^ | HR: 2.13 (1.16; 3.89)^c^ |
| Reference | Professionals |  | 77 573^a^ | 26 (0.034 %)^a^,  IR: 4 (2; 8)^b^ |  |
| **Vingard 1991** (CS) | | | | | |
| Exposure | Smiths and metal processing workers and furnacemen | Hospitalization due to HOA | 5112 | 27 (0.53 %)^a^ | RR: 1.50 (0.90; 1.99)*^b^ |
| Reference | Low exposure blue-collar workers |  | 91 057 | 320 (0.35 %)^a^ |  |
| **Vingard 1992** (CCS) | | | | | |
| Exposure | metal worker | Disability pension due to HOA | n.d. | 15 | RR: 1.8 (0.9; 3.8)*^b^ |
| Reference | Low-exposure persons |  | n.d. | n.d. |  |
| Exposure | metal worker | Disability pension due to low back disorders | n.d. | 121 | RR: 2.6 (1.5; 4.3)^b^ |
| Reference | Low-exposure persons |  | n.d. | n.d. |  |
| *best correspondence to describe the association between occupations in metal work and HOA  a: calculated by the authors  b: adjusted for age (1 quality point)  c: adjusted or matched for age and other important confounder (2 quality points)  CCS: case-control study; CI: confidence interval; CS: cohort study; HOA: Hip osteoarthritis; HR: Hazard ratio; IR: incidence rate; N: number of persons; n.d.: no data; n: number of cases; OR: odds ratio; PY: person years; QS: quality score; RR: relative risk; THR: hip replacement therapy | | | | | |

## Sales

*Table S13: Summary of studies reporting results on the association of occupations in sales and the risk to develop hip osteoarthritis (HOA)*

| **Study** | **Occupational subgroups** | **Outcome** | **N** | **n (%), IR (95%CI) per 100.000 PY** | **Effect (95 %-CI)** |
| --- | --- | --- | --- | --- | --- |
| **Croft 1992a** (CCS) | | | | | |
| Exposure | Warehousemen | THR for HOA or a joint space ≤ 2.5 mm | 53^a^ | 26 (49.1 %)^a^ | OR: 1.1 (0.6; 1.9)^b^ |
| Reference | All other men |  | 486^a^ | 219 ^a^ (45.1 %)^a^ |  |
| Exposure | Warehousemen | THR for HOA or a minimal joint space ≤ 1.5 mm | 53^a^ | 6 (11.3 %)^a^ | OR: 1.3 (0.5; 3.5)*^b^ |
| Reference | All other men |  | 486 ^a^ | 239^a^ (49.2 %)^a^ |  |
| Exposure | Clerks | THR for OA or a joint space ≤ 2.5 mm | 87^a^ | 38 (43.7 %)^a^ | OR: 0.9 (0.6; 1.5)^b^ |
| Reference | All other men |  | 452^a^ | 207^a^ (45.8 %)^a^ |  |
| Exposure | Clerks | THR for HOA or a minimal joint space ≤ 1.5 mm | 87^a^ | 9 (10.3 %)^a^ | OR: 1.1 (0.5; 2.4)^b^ |
| Reference | All other men |  | 452^a^ | 236^a^ (52.2 %)^a^ |  |
| Exposure | Shop worker | THR for HOA or a joint space ≤ 2.5 mm | 59^a^ | 28 (47.5 %)^a^ | OR: 1.2 (0.7; 2.1)^b^ |
| Reference | All other men |  | 480^a^ | 217^a^ (45.2 %)^a^ |  |
| Exposure | Shop worker | THR for HOA or a minimal joint space ≤ 1.5 mm | 59^a^ | 3 (5.1 %)^a^ | OR: 0.6 (0.2; 2.1)^b^ |
| Reference | All other men |  | 480^a^ | 242^a^ (50.4 %)^a^ |  |
| **Elsner 1995** (CCS) | | | | | |
| Exposure | Retail workers | HOA | 11 | 5 (45.4 %)^a^ | OR: 0.5 (0.17; 1.59)*^b^ |
| Reference | All other men |  | 218 | 129 (59.2 %)^a^ |  |
| **Franklin 2010** (CCS, QS=5) | | | | | |
| Exposure | Service and shop worker (as salesperson, police officers, catering personal) | THR | n.d. | 28 [hip]  3 [hip and knee] | OR: 2.1 (1.0; 4.2)*^b^ |
| Reference | Manager and professionals |  | n.d. | 27 [hip]  3 [hip and knee] |  |
| **Solovieva 2018** (CS) | | | | | |
| Exposure | shop worker | Full disability retirement due to HOA | 13 216^a^ | 24 (0.18 %)^a^,  IR: 22 (10; 53)^b^ | HR: 2.35 (1.19; 4.67)*^c^ |
| Reference | Professionals |  | 77 573^a^ | 26 (0.034 %)^a^,  IR: 4 (2; 8)^b^ |  |
| **Vingard 1991** (CS) | | | | | |
| Exposure | Store and warehouse worker | Hospitalization due to HOA | 6144 | 30 (0.49 %)^a^ | RR: 1.39 (0.87; 1.85)*^b^ |
| Reference | Low exposure blue-collar workers |  | 91 057 | 320 (0.35 %)^a^ |  |
| *best correspondence to describe the association between occupations in sales and HOA  a: calculated by the authors  b: adjusted for age (1 quality point)  c: adjusted or matched for age and other important confounder (2 quality points)  CCS: case-control study; CI: confidence interval; CS: cohort study; HOA: Hip osteoarthritis; HR: Hazard ratio; IR: incidence rate; N: number of persons; n.d.: no data; n: number of cases; OR: odds ratio; PY: person years; QS: quality score; RR: relative risk; THR: hip replacement therapy | | | | | |

## Gastronomy

*Table S14: Summary of reported results on the association of occupations in gastronomy and the risk to develop hip osteoarthritis (HOA)*

| **Study** | **Occupational subgroups** | **Outcome** | **N** | **n (%), IR (95%CI) per 100.000 PY** | **Effect (95 %-CI)** |
| --- | --- | --- | --- | --- | --- |
| **Elsner 1995** (CCS) | | | | | |
| Exposure | Employment in gastronomy or hotels | HOA | 14 | 7 (50.0 %)^a^ | OR: 0.6 (0.18; 2.03)^b^ |
| Reference | All other women |  | 215 | 127 (59.1 %)^a^ |  |
| **Solovieva 2018** (CS) | | | | | |
| Exposure | Kitchen workers, building caretakers, cleaners and assistant nurses | Full disability retirement due to HOA | 16 664^a^ | 56 (0.336 %)^a^,  IR: 43 (26; 75)^b^ | HR: 2.58 (1.39; 4.80)^c^ |
| Reference | Professionals |  | 77 573^a^ | 26 (0.034 %)^a^,  IR: 4 (2; 8)^b^ |  |
| **Tüchsen 2003** (CS) | | | | | |
| Exposure | Waiters and bartenders | Hospitalisation due to THR due to HOA | 3496 | 11 (0.31 %)^a^ | SHR: 2.04 (1.02; 3.66)^b^ |
| Reference | All gainfully employed men |  | n.d. | n.d. |  |
| **Vingard 1991** (CS) | | | | | |
| Exposure | Waiters and hairdressers | Hospitalization due to HOA | 2542 | 8 (0.31 %)^a^ | RR: 0.89 (0.42; 1.69)^b^ |
| Reference | Low exposure blue-collar workers |  | 91 057 | 320 (0.35 %)^a^ |  |
| a: calculated by the authors  b: adjusted for age (1 quality point)  c: adjusted or matched for age and other important confounder (2 quality points)  CCS: case-control study; CI: confidence interval; CS: cohort study; HOA: Hip osteoarthritis; HR: Hazard ratio; IR: incidence rate; N: number of persons; n: number of cases; OR: odds ratio; PY: person years; QS: quality score; RR: relative risk | | | | | |

## Food production

*Table S15: Summary of reported results on the association of occupations in food production and the risk to develop hip osteoarthritis (HOA)*

| **Study** | **Occupational subgroups** | **Outcome** | **N** | **n (%)** | **Effect (95 %-CI)** |  |
| --- | --- | --- | --- | --- | --- | --- |
| **Elsner 1995** (CCS) | | | | | |  |
| Exposure | Butcher and baker | HOA | 7 | 5 (71.4 %)^a^ | OR: 1.2 (0.27; 5.62)^b^ | |
| Reference | All other men |  | 222 | 129 (58.1 %)^a^ |  |  |
| **Tüchsen 2003** (CS) | | | | | |  |
| Exposure | Baker, pastry cook and confectionery maker | Hospitalisation due to THR due to HOA | 4487 | 20 (1.0 %)^a^ | SHR: 2.04 (1.25;3.15)^b^ |  |
| Reference | All gainfully employed men |  | n.d. | n.d. |  |  |
| **Vingard 1991** (CS) | | | | | |  |
| Exposure | Mill workers, meat preparer and butcher | Hospitalization due to HOA | 1967 | 15 (0.76 %)^a^ | RR: 2.17 (1.29; 3.65)^b^ |  |
| Reference | Low exposure blue-collar workers |  | 91 057 | 320 (0.35 %)^a^ |  |  |
| **Vingard 1992** (CCS) | | | | | |  |
| Exposure | Food processing worker | Disability pension due to low back disorders | n.d. | 26 | RR: 4.6 (1.1-19.5)^b^ |  |
| Reference | Low-exposure persons |  | n.d. | n.d. |  |  |
| a: calculated by the authors  b: adjusted for age (1 quality point)  CCS: case-control study; CI: confidence interval; CS: cohort study; HOA: Hip osteoarthritis; N: number of persons; n.d.: no data; n: number of cases; OR: odds ratio; QS: quality score; RR: relative risk; SHR: standardized hospitalization ratio (observed / expected number); THR: hip replacement therapy | | | | | |  |

## Storage and transportation

*Table S16: Summary of reported results on the association of occupations in storage and transportation and the risk to develop hip osteoarthritis (HOA)*

| **Study** | **Occupational subgroups** | **Outcome** | **N** | **n (%)** | **Effect (95 %-CI)** |
| --- | --- | --- | --- | --- | --- |
| **Elsner 1995** (CCS) | | | | | |
| Exposure | Postmen | HOA | 7 | 5 (71.4 %)^a^ | OR: 2.2 (0.29; 16.74)*^b^ |
| Reference | All other men |  | 222 | 129 (58.1 %)^a^ |  |
| **Vingard 1991** (CS) | | | | | |
| Exposure | Postmen | Hospitalization due to HOA | 3096 | 10 (0.32 %)^a^ | RR: 0.92 (0.51; 1.81)*^b^ |
| Exposure | Docker and freight handler |  | 1228 | 9 (0.73 %)^a^ | RR: 2.08 (0.92; 3.48)*^b^ |
| Reference | Low exposure blue-collar workers |  | 91 057 | 320 (0.35 %)^a^ | 1 |
| **Vingard 1992** (CCS) | | | | | |
| Exposure | Postmen | Disability pension due to HOA | n.d. | 7 | RR: 2.6 (0.9; 7.9)*^b^ |
| Reference | Low-exposure persons |  | n.d. | n.d. |  |
| Exposure | Storage worker |  | n.d. | 6 | RR: 1.9 (0.6; 5.8)*^b^ |
| Reference | Low-exposure persons |  | n.d. | n.d. |  |
| Exposure | Transport worker |  | n.d. | 8 | RR: 0.9 (0.4; 2.1)^b^ |
| Reference | Low-exposure persons |  | n.d. | n.d. |  |
| Exposure | Transport worker | Disability pension due to low back disorders | n.d. | 70 | RR: 1.3 (0.8; 2.2)^b^ |
| Reference | Low-exposure persons |  | n.d. | n.d. |  |
| *best correspondence to describe the association between occupations in storage and transportation and HOA  a: calculated by the authors  b: adjusted for age (1 quality point)  CCS: case-control study; CI: confidence interval; CS: cohort study; HOA: Hip osteoarthritis; HR: Hazard ratio; N: number of persons; n.d.: no data; n: number of cases; OR: odds ratio; QS: quality score; RR: relative risk; THR: hip replacement therapy | | | | | |

## Health Care

*Table S17: Summary of reported results on the association of occupations in health care and the risk to develop hip osteoarthritis (HOA)*

| **Study** | **Occupational subgroups** | **Outcome** | **N** | **n (%), IR (95%CI) per 100.000 PY** | **Effect (95 %-CI)** |
| --- | --- | --- | --- | --- | --- |
| **Andersen** **2012** (CS) | | | | | |
| Exposure | Health-care assistance | HOA | 84 898^a^ | 299 (0.35 %)^a^, IR: 75,6^b^ | HR: 1.11 (0.99; 1.25)^b^ |
| Reference | Office worker |  | 316 543^a^ | 1473 (0,47 %)^a^, IR: 60,2^b^ |  |
| **Solovieva 2018** (CS) | | | | | |
| Exposure | Nurses and environmental officers | Full disability retirement due to HOA | 57 46^a^ | 11 (0.19 %)^a^,  IR: 24 (8; 78)^b^ | HR: 3.80 (1.78; 8.10)^c^ |
| Reference | Professionals |  | 77 573^a^ | 26 (0.034 %)^a^,  IR: 4 (2; 8)^b^ |  |
| **Tüchsen 2003** (CS) | | | | | |
| Exposure | Medical doctors | Hospitalisation due to THR due to HOA | 8959 | 11 (0.12 %)^a^ | SHR: 0.46 (0.22;0.82)^b^ |
| Reference | All gainfully employed men |  | n.d. | n.d. |  |
| a: calculated by the authors  b: adjusted for age (1 quality point)  c: adjusted or matched for age and other important confounder (2 quality points)  CCS: case-control study; CI: confidence interval; CS: cohort study; HOA: Hip osteoarthritis; HR: Hazard ratio; IR: incidence rate; N: number of persons; n.d.: no data; n: number of cases; OR: odds ratio; PY: person years; QS: quality score; RR: relative risk; THR: hip replacement therapy | | | | | |

## Unskilled and basic level labour

*Table S18: Summary of reported results on the association of unskilled and basic level labour and the risk to develop hip osteoarthritis (HOA)*

| **Study** | **Occupational subgroups** | **Outcome** | **N** | **n (%), IR (95%CI) per 100.000 PY** | **Effect (95 %-CI)** |
| --- | --- | --- | --- | --- | --- |
| **Franklin 2010** (CCS) | | | | | |
| Exposure | Operator and unskilled labour (building construction labourer, heavy truck and lorry driver, cleaner, factory work) | THR | n.d. | 51 (only hip)  6 [hip and knee] | OR: 1.4 (0.78; 2.6)^b^ |
| Reference | Manager and professionals |  | n.d. | 27 [hip]  3 [hip and knee] |  |
| **Solovieva 2018** (CS) | | | | | |
| Exposure | Unskilled transport, construction, and manufacturing workers | Full disability retirement due to HOA | 18 388^a^ | 86 (0.47 %)^a^,  IR: 45 (29; 75)^b^ | HR: 2.94 (1.58; 5.47)^c^ |
| Reference | Professionals |  | 77 573^a^ | 26 (0.034 %)^a^,  IR: 4 (2; 8)^b^ |  |
| **Vingard 1991** (CS) | | | | | |
| Exposure | Unskilled manual workers | Hospitalization due to HOA | 885 | 243 (27.5 %)^a^ | RR: 1.75 (0.99; 2.28)^b^ |
| Reference | Low exposure blue-collar workers |  | 91 057 | 320 (0.35 %)^a^ |  |
| a: calculated by the authors  b: adjusted for age (1 quality point)  c: adjusted or matched for age and other important confounder (2 quality points)  CCS: case-control study; CI: confidence interval; CS: cohort study; HOA: Hip osteoarthritis; HR: Hazard ratio; IR: incidence rate; N: number of persons; n.d.: no data; n: number of cases; OR: odds ratio; PY: person years; QS: quality score; RR: relative risk; THR: hip replacement therapy | | | | | |
